# Bacteriophages as a Strategy to Protect Potato Tubers against *Dickeya dianthicola* and *Pectobacterium carotovorum* Soft Rot

**DOI:** 10.3390/microorganisms10122369

**Published:** 2022-11-30

**Authors:** Filip Beňo, Iveta Horsáková, Martin Kmoch, Karel Petrzik, Gabriela Krátká, Rudolf Ševčík

**Affiliations:** 1 Department of Food Preservation, Faculty of Food and Biochemical Technology, University of Chemistry and Technology, Prague, 166 28 Prague, Czech Republic; 2 Department of Genetic Resources, Laboratory of Virology, Potato Research Institute Havlíčkův Brod, 580 01 Havlíčkův Brod, Czech Republic; 3 Institute of Plant Molecular Biology, Biology Centre of the Czech Academy of Sciences, 370 05 České Budějovice, Czech Republic

**Keywords:** phage control, *Dickeya dianthicola*, *Pectobacterium carotovorum*, soft rot, potato tubers

## Abstract

The protective effect of bacteriophage suspensions (Ds3CZ + Ds20CZ and PcCB7V + PcCB251) on phytopathogenic bacteria causing soft rot of potato tubers, namely *Dickeya dianthicola* (D50, D200) and *Pectobacterium carotovorum* (P87, P224), was observed in ex vivo and in vitro experiments. Ex vivo tests were performed (with air access) on potato slices, on cylindrical cuts from the center of the tubers, and directly in whole potato tubers. In vitro experiments were carried out in a liquid medium using RTS-8 bioreactors, where bacterial growth was monitored as optical density. In particular, the inhibitory effects of phages were confirmed in experiments on potato slices, where suppression of rot development was evident at first glance. Phage treatment against selected bacteria positively affected potato hardness. Hardness of samples treated with bacteria only was statistically significantly reduced (*p* < 0.05 for D50 and *p* < 0.001 for D200 and P87). Ex vivo experiments confirmed significant inhibition of P87 symptom development, partial inhibition of D200 and D50 in phage-treated tubers, and no effect was observed for P224. The inhibitory effect of phages against bacteria was not observed in the in vitro experiment.

## 1. Introduction

Bacterial infection of potato tubers (*Solanum tuberosum* L.) can occur mainly through the disruption of the tissue. The risk of mechanical damage accompanies the entire potato processing technology, from cultivation to storage or sale. The infection of potato tubers by soft rot bacteria starts in the lenticels, at the end of the stolon, or in the surface wounds of the tubers under moist conditions. Tubers can already be infected with the bacteria during harvesting, when the surface of individual tubers is disturbed, during sorting, further handling, and storage. Crop rotation in fields is important to prevent the spread of harmful agents [1,2,3,4,5].

Potato bacterial rot is most commonly caused by bacteria from the *Enterobacteriaceae* family of the genera *Pectobacterium* and *Dickeya*, which were previously taxonomically classified in the genus *Erwinia*. They are also often referred to as bacteria of the Soft Rot *Pectobacteriaceae* (SRP) [6,7]. These bacterial rots cause extensive damage to crops in agriculture and, in the potato sector, a total of EUR 46 million of annual loss in the European Union [8]. In addition to potatoes, they also infect other agriculturally important crops (tomatoes, corn, cabbage, carrots, peppers, beets, etc.) [9,10]. SRP cause both soft rot and a disease called “blackleg” that means blackening of the stem [11]. These bacteria produce specific pectolytic enzymes that break down the cell walls, thus, infecting the plant tissue and obtaining nutrients [12,13].

The blackening of the stem is caused by necrosis of the stems and vascular bundles of the plant under moist conditions. The stem pith then begins to decompose, acquiring the black color from which the rot gets its name [4,14,15]. The disease then spreads from the mother tuber to the whole plant and new parts of the plant. The infected crops then usually produce no or very few new tubers. Any new tubers that appear will usually decompose before harvest. In dry environments, rot causes yellowing of the leaves and wilting of the whole plant, often to the point of crop death [14,15].

Soft rot occurs when the pathogen enters the tuber. The inner surface of the tuber begins to decompose, but the periderm remains intact [4,14]. It causes the most damage during the storage of already harvested tubers. Thus, soft rot increases the risk of spreading the infection, especially in inadequately ventilated cool areas. At a more advanced stage of rot, tuber juices are released into the environment of the infected tubers, through which the bacteria spread to uninfected potatoes, and big pockets of rot are formed [16].

Biological treatments are an important alternative to physical and chemical methods. These methods include the application of bacteria that can produce antimicrobial agents and inhibit the secretion of pathogen enzymes or obligate and facultative predators can be used. Furthermore, plant extracts or bacteriophages can be used as biological crop treatments [1,17]. Similarly, essential oils can be used to reduce bacterial rot damage and extend the shelf life of potato tubers [18]. However, despite their high effectiveness, essential oils are not widely used. The problem is mainly the high volatility and low stability (light, especially UV radiation, etc.), as well as the negative impact on organoleptic properties and phytotoxic effects [19,20,21].

The most important advantage of using bacteriophages in agriculture is their high specificity. Due to this fact, their use in food is safe because they cannot infect humans or animals [1,22]. They replicate very efficiently in the environment and persist as long as their hosts are present. Due to the rapid multiplication of the population, they do not need to be applied repeatedly. Bacteria are able to develop resistance to phages due to competitive interactions between phages and bacteria, similar to antibiotics [23]. There are several mechanisms by which bacteria defend themselves against phage infection; some are species or strain specific, and others are more widespread. Phages, unlike antibiotics, contain genetic information and can assimilate to host defense mechanisms and/or use their own defense systems to break the host’s mechanism [24,25]. The use of phages in plant protection is also limited by phage sensitivity to UV light and low environmental humidity [26]. According to Czajkowski et al. (2017), phages effective against *D. solani* maintain their viability on the tuber surface for several weeks [27].

Preparations can be prepared as a mixture of different bacteriophages for a wider range of host strains [23]. Since bacteriophages are obligate parasites, they naturally co-occur with their hosts. For this reason, phages can be isolated relatively easily from plants affected by bacteria phytopathogens. They are isolated together with their hosts. For example, the phage isolates LIMEstone1 and LIMEstone2, whose host is the bacterium *D. solani*, have been isolated in this way. In a study by Adriaenssens et al. (2012), the effects of the aforementioned isolates were investigated in laboratory and field experiments [28]. The phages reduced the incidence and severity of the disease in vitro and, in field trials, the treatment with the phages resulted in higher tuber yield. Furthermore, phages were isolated from soil samples and their specificity was determined by host range experiments [29]. All phages isolated in this way were specific to *Dickeya* spp.

In this experiment, the inhibitory effect of bacteriophage suspensions used to treat potato tubers inoculated with *Dickeya dianthicola* (D50, D200) and *Pectobacterium carotovorum* (P87, P224) was assessed. To demonstrate the inhibitory effect of the bacteriophages on the growth of the selected bacteria, the culture method was chosen by culture in pits excavated in potato slices and dipping the cylindrical cuts in a solution of bacteria or bacteria and phages. Culture tests were also carried out in whole tubers. Visual aspects, texture (hardness), color, and growth curves were evaluated.

## 2. Materials and Methods

### 2.1. Potatoes (Samples)

Potatoes (*Solanum tuberosum*) grown in the Czech Republic (variety Jindra) were used to verify the inhibitory effect of the bacteriophages used. The potato tubers were grown and harvested according to European regulations. After harvesting, the potatoes were not treated in any way prior to laboratory testing. No disease symptoms were observed on the plants in the field. Before each experiment, potato tubers were surface treated (disinfected) with 3% sodium hypochlorite.

### 2.2. Bacteriophage Suspensions

Two stabilized buffered (50 mM Tris 7.4, 100 mM NaCl, 8 mM MgSO_4_) sterile phage solutions were prepared. The first solution contained (in a 1:1 ratio) *Dickeya* viruses φDs3CZ and φDs20CZ (genus *Limestonevirus*, family *Ackermannviridae*) isolated in the Czech Republic [30] each at a concentration of 10^8^ PFU/mL (plaque forming units/mL). The solution thus prepared was diluted 100-fold to a functional concentration of 10^7^ PFU/mL. Similarly, a second equally concentrated solution was prepared by mixing (1:1) *Pectobacterium* viruses φPcCB7V (genus *Certrevirus*, subfamily *Vequintavirinae*) and φPcCB251 (genus *Berlinvirus*, family *Autographiviridae*) isolated in the Czech Republic [31]. Suspensions containing bacteriophages were stored at 4 °C.

### 2.3. Storage and Preparation of Microbial Suspensions

For use in the experiments, the phage preparations were ten-fold diluted by adding 1 mL of phage suspension (10^8^ PFU/mL) to 9 mL of saline. The saline solution was prepared by dissolving 8.5 g of NaCl (Penta; Prague, Czech Republic) and 1 g of peptone (Merck KGaA; Darmstadt, Germany) in 1000 mL of distilled water and followed by autoclaving.

*Dickeya* spp. strains (CPPB-050, CPPB-200) and *Pectobacterium* spp. strains (CPPB-087, CPPB-224) (Research Institute of Plant Production, v. v. i., Prague-Ruzyně) were amplified on sterile potato dextrose agar (PDA) or in broth (PDB) (Merck KGaA; Darmstadt, Germany). The multiplication of bacteria was performed by removing the grown colony into potato dextrose broth (PDB) (Merck KGaA; Darmstadt, Germany). Bacterial suspensions were cultured at 25 °C and fresh suspensions were prepared at 4.0 MFU (McFarland units) for each experiment.

### 2.4. Cultivation in Dents in Potato Slices

The potatoes were washed, dried, and cut into approximately 1 cm thick slices. Using a circular stainless-steel knife (diameter 1 cm), three dents (0.5 cm deep) were made in each slice near the peel. Then each potato slice was placed separately in a Petri dish. A 100 µL inoculum of the given bacterium was inoculated into dents: *D. dianthicola* (D50), *D. dianthicola* (D200), *P. carotovorum* (P87), or *P. carotovorum* (P224). Similarly, the volume of the phage inoculum was 100 µL. There were six Petri dishes per bacterial strain. Three dents were inoculated with bacteria only and three with bacteria followed by 10^7^ PFU/mL of bacteriophage suspension. Slices inoculated in this way were sealed using parafilm in the Petri dishes and incubated at 25°C inspected after 24, 48, 72, 96, and 168 h. The area infected by soft rot was quantified by image analysis (pixelation).

### 2.5. Cultivation by Soaking Cylinder Cuts

The second method to test the inhibitory effects of phages on bacterial growth was cultivation on cylindrical potato cuts sealed in bags. The potatoes were washed and dried. Cylindrical cuts were made in potato tubers using a 1.5 cm diameter stainless-steel circular knife. These were then cut into 3 cm lengths, washed with distilled water, dried on filter paper, and packed in polymer bags. The samples (cuts) were divided into bags of 6 pieces. The experiment was performed in six parallel repetitions, with a total of 12 bags per bacterium. The first half was inoculated with bacteria only and the second half with bacteria and phages. The prepared bags were placed for cultivation in a thermostat at 25 °C. Selected potato quality parameters were evaluated after 24, 48 and 72 h. Control samples were evaluated at the same time intervals. The effect of phages on the selected bacteria was evaluated by the change in texture (hardness) of potato tubers, the color change, and the effect of phage suspensions on the growth curves (in vitro) of individual bacteria.

### 2.6. Cultivation in Liquid Broth

Bacterial growth in the presence or absence of phages was monitored and evaluated using the Biosan RTS-8 bioreactor (Biosan; Riga, Latvia). Approximately 15 mL of PDB was transferred to 50 mL centrifuge tubes, into which 100 µL of bacterial suspension (1.2 × 10^9^ CFU/mL) with/without 1000 µL of bacteriophages (10^7^ PFU/mL) (multiplicity of infection, MOI = 0.1) was added. Thus, the measurements were carried out in two parallel measurements, with one tube containing the selected bacterial strain and the other tube containing the appropriate bacteriophages added. The tubes thus prepared were placed in a bioreactor and cultured at 25 °C with a speed of 1000 rpm for approximately one week, depending on the particular growth process.

### 2.7. Whole Tuber Cultivation

The inhibitory effect of 100 µL (10^7^ PFU/mL) bacteriophages (MOI = 0.01) on bacterial growth inside potato tubers was evaluated by excavating a hole in the core of the tuber with a 0.6 cm diameter corkscrew and then inoculating the tubers with bacteria or bacteria and phages. This part of the experiment was conducted on only one set of samples, in order to give the reader an idea of the development of soft rot in the potato tuber. Incubation was carried out for one week at 25 °C. After 7 days, the potatoes were cut into half and the extent of bacterial rot was visually assessed. The area infected by soft rot was quantified by image analysis (pixelation).

### 2.8. Texture Measurement

The change in texture during the bacterial rot development was measured instrumentally using an Instron Model 5544 (Instron Ltd.; High Wycombe, Great Britain) instrument via the penetration method in cylindrical samples (cut 1.5 cm diameter and 1.5 cm in length). A 2 mm diameter probe with a penetration rate of 80 mm/min was used to penetrate the samples. A minimum of eight maximum penetration force (N) values was measured for each sample. The measured data were then evaluated statistically and their arithmetic means were plotted on graphs.

### 2.9. Color Measurement

During the development of bacterial rot, color change was monitored in samples by measuring reflectance in the 360–740 nm color spectrum on a Minolta CM-5 spectrophotometer (Konica Minolta; Tokyo, Japan) and processed by SpektraMagic NX Pro (Konica Minolta; Tokyo, Japan). The color values were given by the CIE-L*a*b* color space, which is typically an imaginary color space created mathematically. The values indicate:

L*–the color coordinate between black (0%) and white (100%).

a*–coordinates of the color between red (−values) and green (+values).

b*–coordinates of the color between yellow (−values) and blue (+values).

Measurements were taken at the standard viewing angle (10°), with a daylight source D65, through a 3 mm slit and at laboratory temperature. The instrument was calibrated before each measurement (white and black colors). Due to non-significant differences within parallel samples on the same day, measurements were always made in four bags, two inoculated with bacteria only and two inoculated with phage as well. Ten values were measured for each bag. Because the samples were in the package, the corrected SCI (specular component included) reflectance was observed for the L*, a*, b*, and color spectrum values. The final measurement value for each sample was calculated as the average of all measured data, where outliers according to the Dean–Dixon test were not included. The general comparison of the visible color change at the beginning of the experiment and during the development of bacterial rot was evaluated using the value of the color change coefficient Δ*E* (see Equation (1)) according to [32]:

(1) $$\Delta E=\sqrt{{\left(L^{*}-L_{0}^{*}\right)}^{2}+{\left(a^{*}-a_{0}^{*}\right)}^{2}+{\left(b^{*}-b_{0}^{*}\right)}^{2}}$$

where ∆L*, ∆a*, and ∆b* are differences in color parameters between control and PEF-treated samples.

### 2.10. Statistics

The Statistica 12.0 software (StatSoft; Prague, Czech Republic) was used for statistical data analysis. To test the assumption of normality, the Shapiro–Wilk and Dean–Dixon tests were applied to the collected data. Obtained data are presented as mean ± SD (standard deviation). A one-way post hoc analysis of variance (ANOVA) was performed on the collected data to determine if each of the selected variables (penetration force, coordinates L*, a*, and b*) changed during storage. The variable ∆*E* was not included in the ANOVA test because ∆E is strongly dependent on L*, a*, and b*. The HSD Tukey test was then used to compare the data between each day of sample storage. The significance level was set at *p* = 0.05. Similarly, statistics were also performed between phage-treated and non-phage-treated samples to determine if phage treatment affects the above parameters.

## 3. Results and Discussion

**Figure 1:**
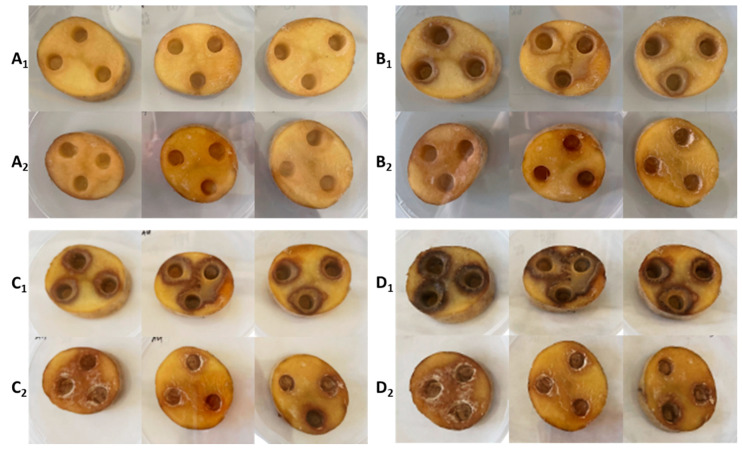
Development of P87 bacterial rot during 96 h at 25 °C on potato slices. (**A_1_**) control at 24 h; (**A_2_**) phage-treated at 24 h; (**B_1_**) control at 48 h; (**B_2_**) phage-treated at 48 h; (**C_1_**) control at 72 h; (**C_2_**) phage-treated at 72 h; (**D_1_**) control at 96 h; (**D_2_**) phage-treated at 96 h.

**Figure 2:**
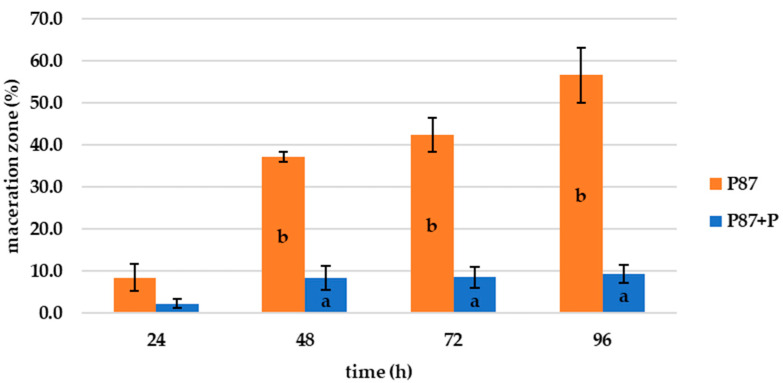
Maceration zones (%) of P87 bacterial rot during 96 h at 25 °C; +P–phage treated; columns represent the means, and the error bars represent the standard deviations; means with different lowercase letters (a or b) are significantly different between the control and phage-treated samples (*p* < 0.05).

The first experiment in this research was to evaluate the effect of bacteriophages on selected potato tuber rot bacteria on potato slices. For this assessment, the method of creating dents in potato slices was used, into which bacteria or bacteria and subsequently (after 10 min) bacteriophages were inoculated. Figure 1 shows the progression of bacterial rot caused by *P. carotovorum* P87 during (index 1) after 96 h compared to potatoes treated with bacteria and bacteriophages (index 2). As can be seen, the phage suspension inhibited the growth of P87 throughout the entire (72 h) storage experiment. In the first 48 h of storage, it is already possible to see the development of soft rot on non-treated potato slices, which accounts for 37.2 ± 1.1% of the surface area (Figure 2). At the end of the experiment, the maceration zone already accounted for 56.6 ± 12.4% of the area, compared to the phage-treated samples. For the slices treated with phage suspensions, the rot formed approximately 9.4 ± 2.2% of the total area during 96 h. While the inhibitory effect of phages on the activity of bacteria can be observed on slices treated with bacteriophage as well, the slices are shriveled but do not show signs of bacterial rot. The visible discoloration of the mesh on the tuber slice in the vicinity of the puncture could be due to oxidation and suberization.

**Figure 3:**
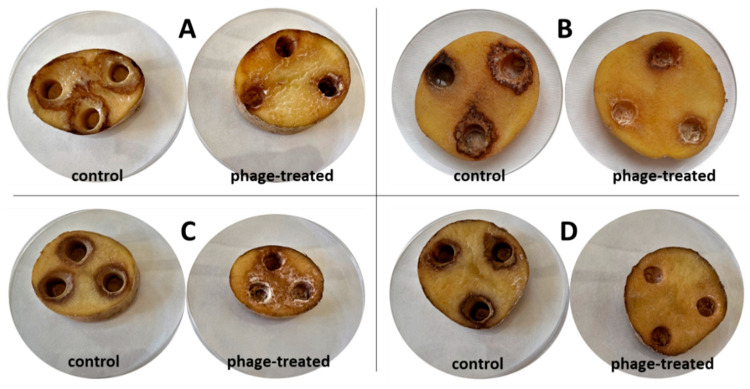
Bacterial rot on potato slices after 72 h of storage at 25 °C; (**A**) D50; (**B**) D200; (**C**) P87; (**D**) P224.

**Figure 4:**
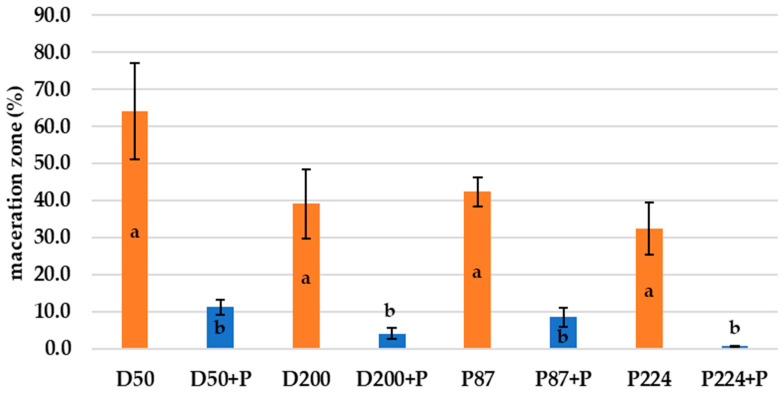
Maceration zones (%) of *D. dianthicola* (D50, D200) and *P. carotovorum* (P87, P224) rots on potato slices after 72 h at 25 °C; +P–phage treated; columns represent the means and the error bars represent the standard deviations; means with different lowercase letters (a or b) are significantly different between the control and phage-treated samples (*p* < 0.05).

Figure 3 shows the growth of different bacterial strain rot after 72 h at 25 °C on slices inoculated with bacterial suspension and bacterial suspension treated with bacteriophages. The visible discoloration of the mesh on the tuber slice in the vicinity of the puncture could be due to oxidation and suberization. However, it is evident from the figure that there was a slowing down and drying of the tuber rot (reduction in the extent of mesh maceration). Statistical evaluation shows a significant improvement in observed parameters between treated/untreated tubers. The most significant increase (72 h at 25 °C) in bacterial rot was observed for *D. dianthicola* D50 (*p* < 0.01), which infected 64.1% of the potato tuber area. In all observed bacterial strains, the macroscopic manifestations of rotting can be observed, with a gradual decomposition of the potato tissue and its discoloration, which is markedly dark at the edges. This discoloration was probably caused due to air access. The positive effect (*p* < 0.05) on preventing rot growth on potato slices was also confirmed by pixelation of soft rot, expressed as maceration zones (%) in Figure 4. Experimental data showed that the mixture of isolated lytic phages at a concentration of 10^7^ PFU/mL effectively suppressed potato soft rot caused by *D. dianthicola* and *P. carotovorum*. Phage-treated samples showed almost no signs of rotting processes. The phage solutions reduced the development of disease symptoms (measured as % of macerated area) by more than 90% or stopped the development completely. These results are consistent with a study by Muturi et al. (2019), which tested the effect of bacteriophages isolated from soil and water samples against the growth of *P. carotovorum* [33]. In their case, soft rot symptoms were reduced by more than 90% when a mixture of bacteriophages at a concentration of 10^9^ PFU/mL was applied. Similarly, Czajkowski et al. (2014) confirmed the inhibitory and lytic effects of bacteriophages on the control bacterium *D. solani* [34].

**Figure 5:**
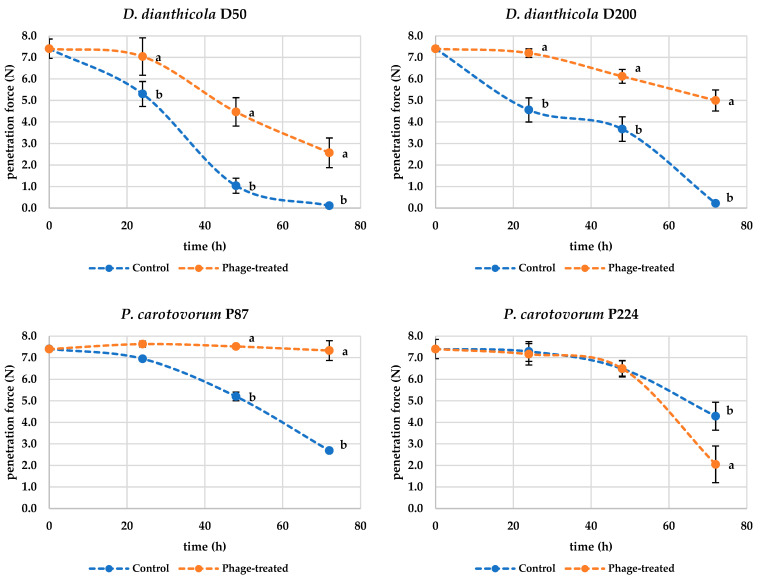
Change in penetrating force (N) during storage of control (inoculated by bacteria) and phage-treated cylinder cuts (*n =* 12); markers represent the means, and the error bars represent the standard deviations; means with different lowercase letters (a or b) are significantly different between the control and phage-treated samples (*p* < 0.05).

The most important characteristic observed in the experiment, where potatoes were inoculated by soaking, was the textural properties (Figure 5) of the samples (hardness). In addition, their color change was also assessed (Table 1). However, these characteristics are not necessarily indicative of the development of bacterial rot.

Figure 5 shows that during the rotting process caused by *D. dianthicola* D50, the textural properties of the potato deteriorate and, therefore, the force required to penetrate the sample decreases. From these observations, it can be concluded that treatment of potato tissue with bacteriophages mitigates the symptoms of rotting by slowing down the decomposition reactions caused by bacterial enzyme-producing pathogens. The measured penetration force data for samples inoculated only with D50 bacteria were statistically different (*p* < 0.001) after 24 h compared to the values measured at the beginning of the experiment, when the penetration force values were F = 7.40 ± 0.45 N for the control samples and F = 5.30 ± 0.58 N for the samples inoculated with D50 bacteria. For the bacteriophage-treated samples, the penetration force value was F = 7.04 ± 0.87 N and was not statistically different compared to the control data (*p* > 0.05) but was statistically different compared to the parallel samples not inoculated with phage (*p* < 0.05). The penetration force measured after 72 h was statistically different (*p* < 0.001) from the values at the beginning of the experiment for both phage-untreated and phage-treated samples; the penetration force for samples inoculated with bacteria was 0.11 ± 0.05 N and for the samples inoculated with both bacteria and bacteriophage was 2.57 ± 0.69 N. The measured values of the experiments were also statistically different (*p* < 0.01) from each other. This fact again confirms that the bacteriophages in this experiment do not explicitly prevent the decomposition of potato tissue but slow down or mitigate the potato decomposition. Similar results were observed for the bacterium D200. The penetration force (7.44 ± 0.45 N in the beginning) gradually decreased more significantly in bacteria-treated potatoes compared to phage-treated potatoes (*p* < 0.001). The hardness of D200-inoculated potatoes after 72 h was then 0.22 ± 0.09 N and phage-treated potatoes 5.00 ± 0.49 N. The bacteria P87 and P224 also showed a statistically significant decrease after 72 h (*p* < 0.01) in penetrating power during the experiment. However, the hardness of the potatoes did not decrease as much as in potatoes inoculated with D50 and D200 bacteria. Similar results to D50 and D200 were observed for P87, where phage treatment positively affected potato texture. However, positive results were not found for bacterium P224. The hardness of the phage-treated potatoes at the end of the experiment was 2.05 ± 0.85 N, which is even lower compared to the control samples, where a penetration force of 4.29 ± 0.65 N was measured. Thus, the results of the penetration force indicate that phages have a positive effect on potato hardness during storage. In all cases, the structure of a potato inoculated with bacteria was destroyed. Unfortunately, it is not possible to compare these results with scientific studies because, to our knowledge, no such studies are available.

**Figure 6:**
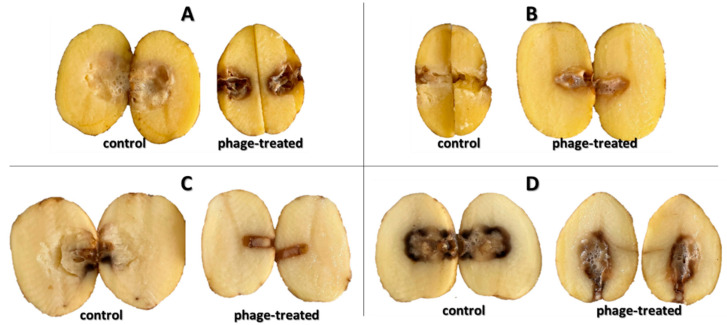
Effect of phage treatment on potato bacterial rots in whole potato tubers after 7 days of storage at 25 °C; (**A**) D50; (**B**) D200; (**C**) P87; (**D**) P224.

**Figure 7:**
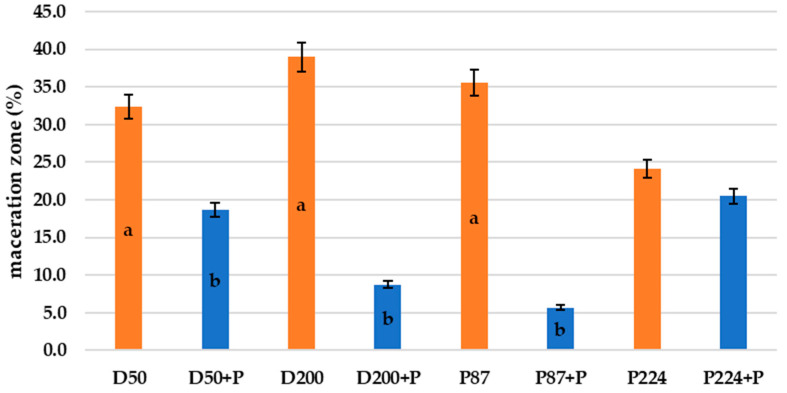
Mesh maceration (%) of *D. dianthicola* (D50, D200) and *P. carotovorum* (P87, P224) rots in whole potato tubers after 7 days at 25 °C; +P–phage treated; columns represent the means and the error bars represent the standard deviations; means with different lowercase letters (a or b) are significantly different between the control and phage-treated samples (*p* < 0.05).

Figure 6 shows the growth of bacterial rot in whole potato tubers, phage treated or non-treated, after 72 h at 25 °C. There is less soft rot (mesh maceration) on phage-treated potato tubers. On these tubers, there is visible discoloration and drying of the rot, which makes the rot darker and more pronounced. The most pronounced inhibitory effect was observed with bacterium P87 (*p* < 0.05), followed by bacteria D200 and D50 (*p* < 0.05). The phage suspension reduced the symptoms of P87 rot by 83.9% and 42.3–77.6% for D50 and D200 bacteria, respectively. The last sample is a potato treated with *P. carotovorum* P224, where no significant differences are visible between phage-treated and untreated tubers (*p* > 0.05). Areas of mesh maceration by individual bacterial rots can be seen in Figure 7. Another option could be to inoculate potatoes by dipping them in appropriate suspensions of bacteria and bacteriophages. Soaking of whole tubers was carried out in a study by Hassan et al. (2017), where out of nine bacteriophage isolates, four showed inhibitory effects against all tested *P. carotovorum* strains [35]. In a study by Bugaeva et al. (2021), they applied phages in the form of regular spraying to potatoes stored in storage and maintained also by conventional measures, such as maintaining temperature and relative humidity [36]. According to their results, by applying bacteriophages, they were able to reduce the concentration of bacteria in tubers, but due to the effectiveness of storage conditions, the study is not completely conclusive in terms of suppression of disease symptoms by bacteriophages. In a study by Zaczek-Moczydłowska et al. (2020), they tested the efficacy of selected phages to inhibit *Pectobacterium* spp. in ex vivo experiments by incubating tubers surface inoculated with bacteria or phages cut in half [37]. For evaluation, they compared the initial tuber weight and the tuber weight at the end of the experiment after removal of the macerated mass.

Based on the determination of the color and the color coefficients L*, a*, and b*, respectively (Table 1), it can be seen that the color of the potatoes changed during the storage experiment. In particular, the most significant changes were observed for the lightness value L*, where there was a significant increase in lightness (*p* < 0.01) in all cases for all tested potatoes treated with both bacteria only and bacteria with phages compared to the control samples at the beginning of the experiment. A similar trend of color changes within the values of parameters a* and b* was also observed. Significant changes were observed, especially for the parameter a* in potatoes treated with D50 bacteria (*p* < 0.05). On the contrary, no significant differences (*p* > 0.05) were observed for the b* parameter in potatoes treated with bacteria P87, respectively, P87 + P. Significant statistical differences in color coordinators, except for L* and a*, were also not detected for P224 during storage. No significant statistical differences were shown between control and P224-treated samples either. From the measured data of color parameters, the color change coefficient ∆*E* was calculated, always between the values at the beginning of the measurement and 24 h of storage, respectively 48 and 72 h. From the measured data of color parameters, the color change coefficient ∆*E* was calculated, always between the values at the beginning of the measurement and 24 h of storage, respectively, 48 and 72 h. As can be seen in Table 1, the color change coefficients also confirm the color change in the potato after treatment during storage. Within 24 h of storage, a color change occurred, with the ∆*E* coefficient for the measured samples ranging between 3.8 and 14.4. Shevell (2003) defined the range of ∆*E* coefficient with a range from 2 to 10 as a color change that can be observed at the first glance of the observer [32] and the range of ∆*E* coefficient range 11–49 as colors similar to their opposite. The ∆*E* coefficients were also calculated to compare the color of potatoes treated with bacteria alone and those treated with bacteria and phages. In this case, the range of ∆*E* was from 1.1 to 6.9. The highest value of the ∆*E* coefficient was calculated for bacterium D50 after 24 h of storage. Again, these values lie in a range of 2 to 10 according to Shevell (2003). Thus, it can be said that there is a color difference between bacteria-treated and phage-treated potatoes.

**Figure 8:**
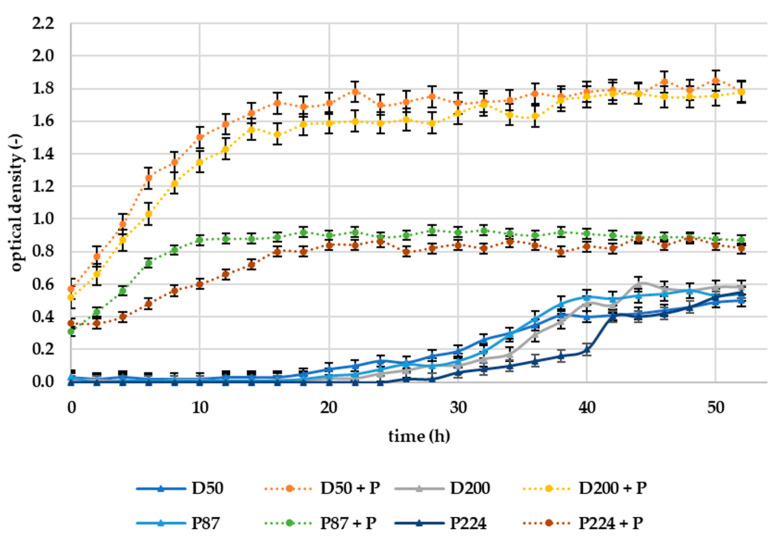
Effect of bacteriophages (+P) on growth curves of *D. dianthicola* (D50, D200) and *P. carotovorum* (P87, P224), in vitro experiment in liquid broth; +P–phage treated; markers represent the moving means and the error bars represent the standard deviations.

The results of the in vitro experiment can be seen in Figure 8. The efficacy of selected lytic phages (10^7^ PFU/mL) on bacteria was assessed in a dynamic environment where the medium was mixed at a rotation of 1000 rpm. The in vitro experiment in liquid medium (tryptic soy broth, TSB) was performed in a laboratory bioreactor RTS-8, evaluating the optical density of the solution (broth).

The results of in vitro measurements in liquid medium suggest opposite effects of phages than experiments directly on potato samples. The addition of bacteriophages to the suspension of all tested bacteria shortens the lag phase of growth and increases the overall bacterial growth, as can be seen in Figure 8. The resulting optical density after 50 h was approximately 3-times higher for D50 and D200 bacteria treated with phages compared to untreated samples. For bacteria P87 and P224, it was then approximately 30% higher. As we can hypothesize from our measured results, bacteriophages are unable to inhibit bacteria in a constantly rotating liquid environment. These results are in line with those obtained by Bartnik et al. (2022), where successful inhibition of *D. solani* was also not confirmed in an in vitro experiment [38]. In a study by Zaczek-Moczydłowska et al. (2020), a bacteriophage mixture was tested in vitro by mixing 100 µL of a bacterial suspension of *Pectobacterium* spp. at a concentration of 10^8^ CFU/mL, with 4 mL of a phage mixture at a concentration of 10^8^ PFU/mL [37]. The growth curves of the bacteria were measured using optical density at 600 nm. Measurements were taken every hour for the first five hours and then after 24 h. As a result, the OD of the bacteriophage-treated samples was reduced, thus confirming the inhibitory effect, even in liquid medium. The study does not elaborate on how the samples were incubated, but it can be assumed that they were in a static environment. This would confirm our hypothesis that bacteriophages are unable to infect bacteria under constant rotation of the system. A similar experiment was performed by Soleimani-Delfan et al. (2015) but against *Dickeya* spp., with the difference that the samples were incubated on a rotary shaker at 120 rpm. Spectrophotometric optical density measurements showed inhibitory effects of bacteriophages [39]. In the current experiment, the rotational speed was much more intense (1000 rpm), so a reduction could lead to more promising results.

## 4. Conclusions

A positive inhibitory effect of bacteriophages against selected phytopathogens was observed in ex vivo experiments. On potato slices, there was a visible inhibition of bacterial rot growth in all cases and, on whole tubers, there was a successful inhibition of bacteria, especially for P87, while no effect was observed against P224. The phage treatment of potatoes also had a positive effect on potato hardness. The hardness of potatoes inoculated with bacteria alone decreased significantly within 72 h. Color changes were also observed during storage, but it is questionable whether this is sufficient evidence of a positive effect of phages against bacteria. Experiments in liquid medium incubated in vitro in the bioreactor indicated opposite effects of phage on bacteria compared to ex vivo experiments. From the above, it appears that effective bacteriophage applications will not include dynamic methods (e.g., washing or floating potatoes). Static application methods, such as sprinkling or spraying treatment with phage suspension, may be more effective.

## Figures and Tables

**Table 1:** Potato cylindrical cut color (Mean ± SD) during storage at 25 °C and values of the color change coefficient Δ*E*.

Bacteria	Parameter	0 h	24 h	∆*E* (0–24 h)	48 h	∆*E* (0–48 h)	72 h	∆*E* (0–72 h)	ANOVA *p*-Value
D50	L*	50.1 ^bcd^ ± 2.5	56.3 ^a^ ± 3.5	14.4	58.7 ^a^ ± 3.8	8.8	60.6 ^a^ ± 3.7	12.3	5.884 × 10^−15^
	a*	–2.4 ^b^ ± 0.4	–1.7 ^aBcd^ ± 0.8		–2.9 ^b^ ± 0.6		–2.8 ^Bb^ ± 0.7		0.0003
	b*	10.6 ^bc^ ± 2.2	12.5 ^aBcd^ ± 5.6		12.5 ^aBbd^ ± 4.1		17.0 ^bBc^ ± 4.4		1.925 × 10^−12^
D50 + P	L*	50.1 ^bcd^ ± 2.5	57.8 ^ad^ ± 3.5	10.0	56.4 ^a^ ± 4.4	6.5	59.3 ^ab^ ± 5.2	10.0	3.331 × 10^−15^
	a*	–2.4 ^b^ ± 0.4	–1.3 ^aAcd^ ± 1.1		–2.4 ^b^ ± 0.4		–2.1 ^Ab^ ± 0.7		2.023 × 10^−5^
	b*	10.6 ^bd^ ± 2.2	16.8 ^aAcd^ ± 3.4		8.6 ^Abd^ ± 1.7		14.5 ^aAbc^ ± 6.8		3.364 × 10^−14^
∆*E* (D50–D50+P)		-	6.9	-	4.7	-	2.9	-	-
ANOVA D50 × D50+P		*p*-value L*	0.8723	-	0.4553	-	0.4557	-	-
		*p*-value a*	4.0267 × 10^−6^		0.0760		0.0079		
		*p*-value b*	0.0003		0.0477		2.2161 × 10^−6^		
D200	L*	48.6 ^bcd^ ± 7.5	57.4 ^a^ ± 5.9	8.9	60.4 ^aB^ ± 3.8	12.0	59.7 ^a^ ± 6.1	11.6	6.140 × 10^−11^
	a*	–2.7 ^b^ ± 0.5	–3.2 ^aBd^ ± 0.9		–3.1 ^Bd^ ± 0.5		–2.6 ^bBc^ ± 0.6		0.0010
	b*	16.6 ^d^ ± 2.8	16.4 ^d^ ± 5.0		14.4 ± 3.2		13.0 ^abB^ ± 3.1		0.0014
D200 + P	L*	48.6 ^bcd^ ± 7.5	55.6 ^ad^ ± 5.2	7.3	57.3 ^aA^ ± 4.9	9.8	62.0 ^ab^ ± 3.8	13.4	1.005 × 10^−11^
	a*	–2.7 ± 0.5	–2.4 ^Ad^ ± 0.6		–2.4 ^A^ ± 0.8		–3.1 ^Ab^ ± 0.6		0.0015
	b*	16.6 ^c^ ± 2.8	14.9 ^c^ ± 2.9		12.2 ^abd^ ± 4.1		16.0 ^Ac^ ± 3.3		3.807 × 10^−5^
∆*E* (D200–D200+P)		-	2.5	-	3.9	-	3.8	-	-
ANOVA D200 × D200+P		*p*-value L*	0.3384	-	0.0380	-	0.1596	-	-
		*p*-value a*	0.0009		0.0049		0.0123		
		*p*-value b*	0.2608		0.0667		0.0080		
P87	L*	48.0 ^bcd^ ± 7.1	55.3 ^a^ ± 4.4	7.7	60.4 ^a^ ± 5.1	12.7	61.3 ^a^ ± 5.8	14.0	3.331 × 10^−16^
	a*	–2.1 ^d^ ± 0.5	–2.3 ± 0.6		–2.4 ± 0.7		–2.9 ^a^ ± 0.8		0.0259
	b*	10.8 ± 3.4	13.0 ± 2.7		13.6 ± 3.4		14.8 ± 3.6		0.3038
P87 + P	L*	48.0 ^bcd^ ± 7.1	54.3 ^acd^ ± 4.4	6.6	58.2 ^ab^ ± 4.7	10.3	57.8 ^ab^ ± 5.3	10.2	1.110 × 10^−16^
	a*	–2.1 ^d^ ± 0.5	–2.3 ^d^ ± 0.5		–2.2 ^d^ ± 0.7		–2.5 ^abc^ ± 0.6		2.897 × 10^−5^
	b*	10.8 ± 3.4	12.5 ± 2.3		11.2 ± 3.2		13.2 ± 3.2		0.1559
∆*E* (P87–D87+P)		-	1.1	-	3.2	-	3.8	-	-
ANOVA P87 × P87+P		*p*-value L*	0.3475	-	0.1445	-	0.1098	-	-
		*p*-value a*	0.1280		0.7593		0.0733		
		*p*-value b*	0.2260		0.3747		0.4715		
P224	L*	57.8 ^c^ ± 2.6	55.3 ^cd^ ± 2.9	4.9	67.9 ^abB^ ± 10.2	11.8	64.8 ^b^ ± 5.2	8.4	5.296 × 10^−5^
	a*	−3.2 ^bd^ ± 0.3	−2.0 ^acd^ ± 0.6		−3.7 ^bd^ ± 0.6		−2.8 ^bc^ ± 0.5		3.780 × 10^−8^
	b*	17.2 ± 2.6	19.9 ^d^ ± 3.0		20.3 ^c^ ± 4.8		15.5 ^bc^ ± 3.1		0.0105
P224 + P	L*	57.8 ^c^ ± 2.6	55.9 ^d^ ± 3.0	3.8	63.8 ^A^ ± 4.9	7.4	59.3 ^b^ ± 7.8	7.5	0.0093
	a*	−3.2 ± 0.3	−2.5 ^c^ ± 0.2		−3.5 ^b^ ± 0.7		−3.0 ± 1.0		0.0112
	b*	17.2 ± 2.6	18.0 ± 1.9		18.0 ± 4.3		17.7 ± 5.8		0.9603
∆*E* (P224–P224+P)		-	4.8	-	9.8	-	12.0	-	-
ANOVA P224 × P224+P		*p*-value L*	0.6625	-	0.0138	-	0.1211	-	-
		*p*-value a*	0.2633		0.4945		0.2715		
		*p*-value b*	0.0639		0.5998		0.3106		

Values followed by a small index in the rows (within the parameters L*, a*, and b*) differ significantly (*p* < 0.05) during storage and values followed by a large index in the columns (within the difference between treated and untreated potatoes) differ significantly (*p* < 0.05) (Tukey HSD test in one-way ANOVA, separately for each bacterial species).

## Data Availability

Data available on request due to privacy restrictions. The data presented in this study are available on request from the corresponding author.
